# Transplantation of Tendon from Animal to Man

**Published:** 1887-06

**Authors:** 


### *TRANSPLANTATION OF TENDON FROM ANIMAL TO MAN.*

The Paris correspondent of the *British Medical Journal*, February 12, 1887, refers to a note by M. Peyrot on the transplantation of tendon from an animal to man. The patient, a boy, aged 14, entered the Hôtel Dieu in October, 1885. Six months previous to that date, he had received a wound on the palmar aspect of the first phalanx of the left middle finger, near the groove between the first and second phalanx. The flexor tendons were completely divided, and the finger was forcibly drawn back by the extensors. Simple suture of the two ends was out of the question, on account of the great length of time that had elapsed since the infliction of the injury. On October 30th an incision was made over the first phalanx in its palmar aspect, extending into the palm of the hand for a distance of about four centimetres. The ends of the tendon having been found with some difficulty, a piece of flexor-tendon, thirty-three millimetres in length, was taken from the hind paw of a young dog, and united to the vitalized ends of the divided tendon by three stitches of catgut. The incision itself was brought together by two rows of stitches; a deep one, near the transplanted tendon, in order to form a sheath, and a superficial one, in the skin. The operation was done with the most minute antiseptic precautions. When the first dressing was removed, ten days after the operation, it was found that there had been no suppuration; but, unfortunately, union had not taken place. There was, however, no sloughing, and the wound healed well. When the patient left the hospital on January 4, 1886, he had regained the use of all the fingers of the injured hand, though the movements were still feeble. In a similar case in a child aged two and a half years, the transplanted tendon (the tendo Achillis of a cat) was completely eliminated three weeks after the operation. M. Peyrot considers that, as a general rule, transplantation should be had recourse to only in cases of real loss of substance, or when the length of time which has elapsed since the injury has produced a definitive separation of the two ends of the tendon.—*Therapeutic Gazette.*

